# Grip Strength-Endurance in Ambitious and Recreational Climbers: Does the Strength Decrement Index Serve as a Feasible Measure?

**DOI:** 10.3390/ijerph17249530

**Published:** 2020-12-19

**Authors:** Berit Kristin Labott, Steffen Held, Lars Donath

**Affiliations:** 1 Institute of Sport Science, Otto-von-Guericke University, Zschokkestr. 32, D-39106 Magdeburg, Germany; berit.labott@ovgu.de; 2 Department of Intervention Research in Exercise Training, Institute of Exercise Training and Sport Informatics, German Sport University Cologne, Am Sportpark Müngersdorf 6, D-50933 Cologne, Germany; s.held@dshs-koeln.de

**Keywords:** hand, force decline, asymmetry, rock, boulder, power

## Abstract

The present study investigated the time course of repetitive maximal isometric grip strength, depending on the arm position, laterality (dominant vs. non-dominant side), and climbing level. The intervention aimed to provide a feasible indicator of maximal strength-endurance in climbing. Seventeen recreational (climbing level (CL): 6.8 (SD 0.5) on the Union Internationale des Associations d’Alpinisme (UIAA) metric scale) and eleven ambitious (CL: 8.7 (SD 0.6) UIAA metric scale) climbers (age: 27 (8) years; BMI: 21.6 (1.9) kg/m^2^; ape index (arm span divided by body height): 1.05 (0.18); training volume: 2.2 (1.0) h/week). Participants completed maximal isometric handgrip strength (F_max_) tests in four positions (left and right hand beside the trunk as well as left and right hand above the shoulder) plus twelve repetitive work-relief cycles, lasting 4 and 1 s where isometric strength, heart rate, and perceived exertion were recorded. F_max_ differed between groups in nearly all positions. A large side × position × time × group interaction was observed for repetitive isometric grip strength (*p* = 0.009, η_p_^2^ = 0.71). However, subsequent post-hoc tests did not reveal a significant difference between groups during each testing position. Additional correlation analysis between asymmetry and CL showed an inverse relationship for ambitious climbers (r = −0.71). In conclusion, the degree of grip strength decline did not relevantly differentiate between ambitious and recreational climbers. Thus, the time course of handgrip strength seems to mainly rely on maximal grip strength during the first contraction.

## 1. Introduction

Recreational, ambitious, and elite sport climbing has gained notable popularity within the last two decades. A strong Olympic movement with its first recognition by the International Olympic Committee (IOC) in 2007 and the ongoing professionalization of sport climbing call for specific and valid measures to objectify individual performance levels in climbing. Such performance-determining parameters may then serve as relevant outcome measures following a progressively conducted climbing training, e.g., for performance monitoring during the training periods of climbers on various level.

Numerous studies in the past intended to derive and assess the performance parameters of climbing to contribute to a comprehensive sport climbing performance structure. A majority of these predominantly cross-sectional studies investigated, for example, aerobic energy costs [1,2], anthropometric determinants [3], anaerobic capacities of the forearm muscles [4], active and passive recovery [5], fingertip force [6], or work–relief ratio of load application [7].

Handgrip measures were also frequently considered in climbing-specific performance structure models. Although handgrip strength has been frequently regarded as a more general measure of local climbing strength mirroring less climbing-specific demands [8], finger and hand muscle strength testing was repeatedly reported to be the performance-limiting factor during climbing [6]. A review by Saul et al. in this regard underlined that the important elements in climbing are hand and forearm strength and endurance, also finding that handgrip strength is favorable for success in sport elite climbers [9]. Well-trained forearm flexors with high-aerobic capacities are thereby essential for an efficient climbing style [9].

Despite the available body of evidence on sex, age, laterality, and climbing level, climbing-specific relevance of different handgrip measures remain elusive. This is of greater importance because handgrip dynamometry does not reflect the heterogeneity of different hold positions [10] and is not specific enough to reflect performance of elite climbers [11].

Therefore, many efforts have been made to modify handgrip measures while considering climbing-specific hold position [6]. However, none of these studies investigated the left- and right-sided time course of handgrip strength in different positions depending on climbing level during a typical work–relief ratio of climbing as a grip strength-endurance surrogate [7,12].

Therefore, the present study investigated differences in maximal handgrip strength and handgrip strength-endurance in two groups of the dominant and non-dominant side during various arm–hand positions. The hypothesis is that the handgrip strength decline differs between ambitious and recreational climbers. The hypothesis was that the force decline is steeper and the maximal grip strength is lower in recreational compared to ambitious climbers. Thus, the intention of this study was to provide data on the time course of grip strength values depending on climbing level and arm position.

## 2. Materials and Methods

### 2.1. Participants

Twenty-eight right-handed young adult sport climbers were included in this study (Table 1). None of the participants reported any acute or chronic cardio-circulatory or metabolic diseases, health complaints, or previous injury. After a comprehensive study instruction, all subjects signed an informed consent to the study. The experimental setting of the study complied with the Declaration of Helsinki 2013. As the data were collected within standard handgrip screening, an ethical approval was obtained from the ethical committee of the German Sports university (127/2020). All included subjects practiced indoor sports climbing for at least one year. As a climbing-specific anthropometric measure, the ape index was calculated as arm span divided by body height [13]. Thereby, the largest tip-to-tip distance between the extended fingers was measured as arm span. Data were collected during upright stance. Climbing level was reported as the best redpoint ascent (complete ascent without falling with previously available information/inspections of the route) within the current year. A climbing level of 8.0 of the Union Internationale des Associations d’Alpinisme (UIAA) metric scale [14] was required to be allocated to the ambitious group.

### 2.2. Testing

Before conducting the handgrip measures, the use of the 6–20-point rating of perceived exertion (RPE) Borg scale was introduced [15], and a heart rate monitor (Accurex Plus^®^, Polar Electro Oy, Kempele, Finland) was attached via a dampened chest strap. By performing some practice trials and hand mobilisations, all participants were able to familiarize themselves with the hydraulic handgrip dynamometer (Saehan^®^, Masan, Korea). The handgrip position had to be comfortable and the fingers needed to fully surround the device so that all the tips of the fingers were on one side [16]. The order of the four arm positions for handgrip testing was inter-individually randomly assigned.

#### Testing Positions

**Figure 1:**
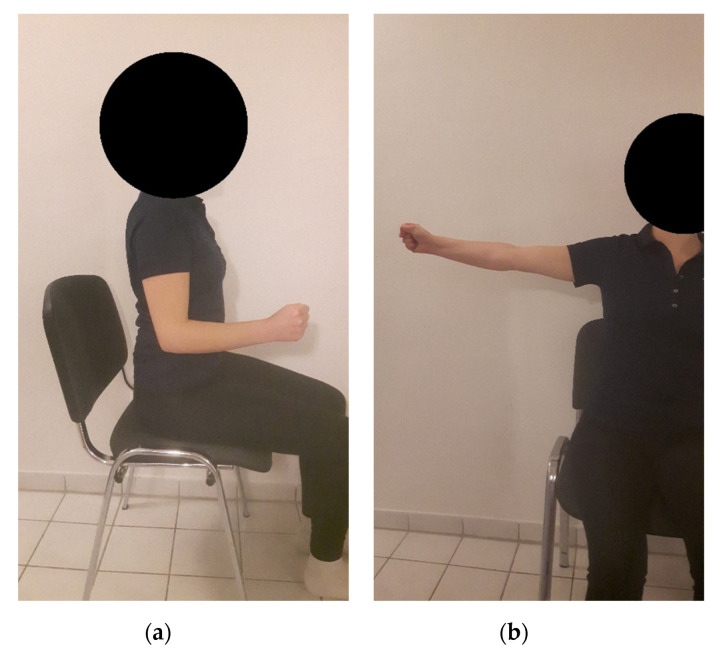
Visualization of testing positions (here demonstrated for the right hand): (**a**) upper arm straight beside the trunk, without abduction or rotation and with an elbow angle of 90°; (**b**) lifted arm position, 90° abducted and 90° external rotation.

In total, four testing positions were applied, two testing positions with the left and two testing positions with the right hand from a seated position: upper arm straight beside the trunk, without abduction or rotation and with an elbow angle of 90°; and, lifted arm position, 90° abducted and 90° external rotation (Figure 1). As mentioned in the testing paragraph, the order of the arm positions was random.

Two sets of the four positions were allowed. The four positions were tested one after the other. A 5 min break between each testing position was guaranteed. Then, all testing positions were measured a second time. Between the first and second rounds of testing, a complete break of 10 min was provided. The best trial of each position was included in further analysis. Before the repetitive strength endurance testing started, maximal isometric handgrip strength was assessed in each of the respective four arm positions. Then, each strength-endurance attempt consisted of 12 consecutive work–relief cycles with maximal effort. In accordance with a climbing-specific strain character, the work period lasted four seconds and the relief phase lasted one second [7]. An acoustic signal indicated the work and relief pattern. Verbal encouragement during the work period was also given by shouting out “go, go, go, go”. Relief was initiated by commanding “relax”. Work and relief periods alternated. The analogue gauge needle amplitude of the handgrip device was recorded by a 100 Hz sampling digital video camera (HandyCam DCR-DVD 201E PAL, Sony, Tokyo, Japan).

### 2.3. Statistical Analysis

The highest peak value within the four second work period was analysed. Force values of the handgrip device are provided in kilograms. The relative force values were adjusted to the individual’s bodyweight in kilograms. Before and after the twelve repetitions, heartrate increases were recorded during each of the four positions. RPE levels were requested after the final repetition. As a measure of handgrip strength-endurance, the strength decrement index (SDI) was assessed according to Jones et al. [17]. Therefore, the first three (mean_first_) and last three (mean_last_) work phases were separately averaged. Then, the percentage decline of handgrip strength was calculated using the following formula: SDI [%] = ((mean_first_ − mean_last_)/ mean_first_)·100.

Statistical analyses were conducted using SPSS 20 (IBM SPSS, Chicago, IL, USA). Data were tested for normal distribution (Kolmogorov–Smirnov test) and homogeneity of variances (Levene test). Demographic baseline data were analysed via multivariate analyses of variances (MANOVA) and follow-up univariate analyses for each parameter.

Differences between the left and right side of the upper and lower hold positions were calculated for maximal grip strength, SDI, and RPE. Therefore, separately 2 (group: recreational vs. ambitious) × 2 (side: left vs. right) repeated measures analyses of variances (rANOVA) were conducted for the upper and lower hold positions.

Concerning the consecutive time course of maximal grip strength, a complex rANOVA was performed. Thereby, a 2 (side: left vs. right) × 2 (position: upper hold vs. lower hold) × 12 (time: 12 grip strength cycles) was calculated. Tukey’s honestly significant difference (HSD) post-hoc tests were applied in case of significant main and interaction effects. This procedure was analogously conducted for heartrate analysis.

Spearman correlation coefficients were calculated between the difference in the right and left side (degree of asymmetry level) during the upper and lower position for recreational and ambitious climbers separately.

To estimate the corresponding main or interaction effect sizes, partial eta squares (η_p_^2^) were additionally calculated. Thereby, η_p_^2^ ≥ 0.01 indicates a small effect, η_p_^2^ ≥ 0.059 a medium effect, and η_p_^2^ ≥ 0.138 a large effect [18]. Cohen’s d effect sizes were provided for pairwise comparison (trivial: d < 0.2, small: 0.2 ≤ d < 0.5, moderate: 0.5 ≤ d< 0.8, large d ≥ 0.8) [19].

## 3. Results

### 3.1. Relative Force

**Figure 2:**
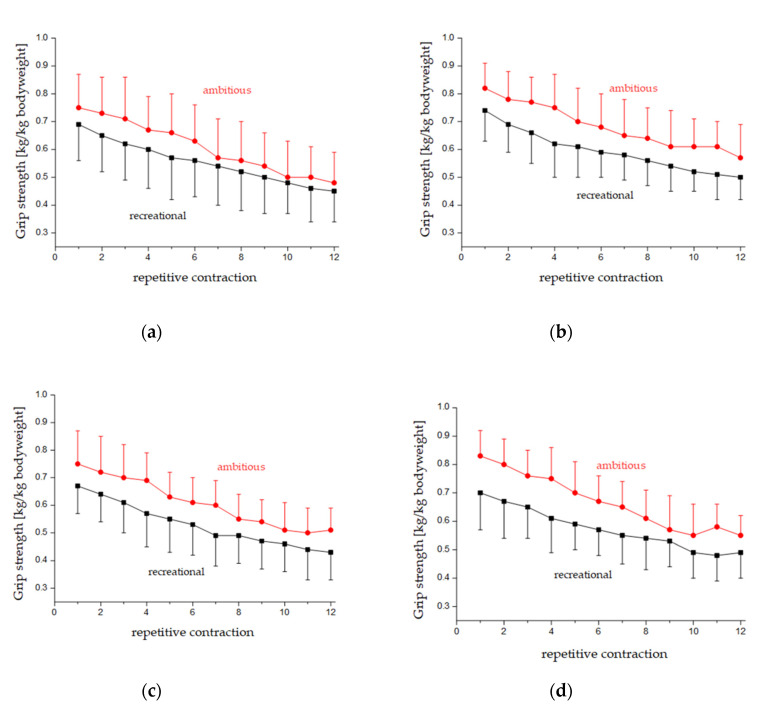
Relative force decline (kg/kg bodyweight) within the 12 repetitive contractions for ambitious (circles) and recreational (squares) climbers during the upper grip positions on the left (**a**) and right (**b**) side as well as the lower positions left (**c**) and right (**d**).

A significant main effect of the factor side was found for both grip positions (lower grip positions: F_1,26_ = 22.7, *p* < 0.001, η_p_^2^ = 0.47, upper grip positions: F_1,26_ = 11.4, *p* = 0.002, η_p_^2^ = 0.30) with higher consecutive strength values on the dominant (right) side. Irrespective of the time course, a side × group interaction effect with a large effect size was found only for lower grip positions (F_1,26_ = 4.6, *p* < 0.04, η_p_^2^ = 0.15). Post-hoc testing for the interaction effect revealed significant differences between the left and right side for both the ambitious (left: 0.76 kg (SD 0.09) vs. right: 0.86 (0.08), *p* = 0.01) and recreation groups (left: 0.69 kg/kg bodyweight (SD 0.12) vs. right: 0.74 (0.09), *p* = 0.04). The upper grip position did not significantly differ for the ambitious (left: 0.74 (SD 0.10) vs. right: 0.84 (0.08), *p* = 0.11) and the recreation groups (left: 0.71 (SD 0.09) vs. right: 0.75 (0.11), *p* = 0.34). (Figure 2).

With notably high effect sizes, significant side effects with higher values on the dominant (right) side (F_1,26_ = 41.5, *p* < 0.001, η_p_^2^ = 0.62) and time effects (F_1,26_ = 44.6, *p* < 0.001, η_p_^2^ = 0.97) were found for the relative force decline (Figure 2). However, interaction effects were not observed (*p* > 0.45).

### 3.2. Strength Decrement Index (SDI)

**Figure 3:**
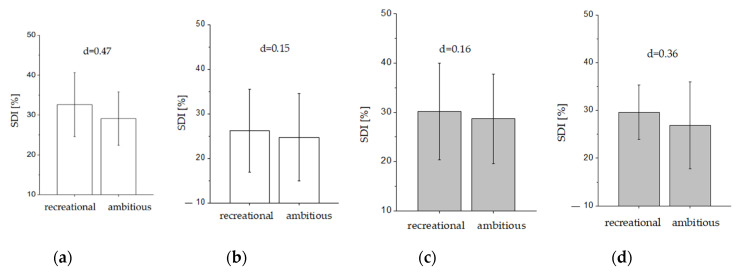
Strength decrement index (SDI) in percent for recreational and ambitious climbers during the upper grip positions on the left (**a**) and right (**b**) side as well as the lower position left (**c**) and right (**d**). Cohen’s d was provided as a measure of effect size estimation between groups for each position (trivial: d < 0.2, small: 0.2 ≤ d < 0.5, moderate: 0.5 ≤ d < 0.8, large d ≥ 0.8).

Concerning percentage SDI, a side effect with a lower strength decline was only observed for the upper grip positions (F_1, 26_ = 7.4, *p* = 0.01, η_p_^2^ = 0.22). The lower grip position did not reveal a side effect (F_1, 26_ = 0.28, *p* = 0.60, η_p_^2^ = 0.01) (Figure 3c,d). Group x side interactions were not observed (*p* > 0.32). Pairwise Cohen’s d effect sizes indicated merely a small effect (0.15 < d < 0.47).

### 3.3. Asymmetry Levels

When comparing asymmetry levels between the left and right side for the upper and lower handgrip position, weak and non-significant inverse correlations were found for recreational climbers (low position, r = −0.05; *p* = 0.67, upper position, r = −0.31; *p* = 0.13). In contrast, a moderate and significant correlation was found between lower position asymmetry levels in ambitious climbers (r = −0.70; *p* = 0.03). For the upper position, a weak and non-significant correlation, similar to the recreational climbers, was found (r = −0.30; *p* = 0.21).

### 3.4. Heartrate

With a significant time × group interaction effect (F1,26 = 6.51, *p* = 0.017, η_p_^2^ = 0.20), heartrates only increased differently between groups from the 1st to the 12th consecutive handgrip trial during the left-sided position bottom beside the body (ambitious, pre: 91 min−1 (SD 19) to post: 106 (24); recreational, pre: 91 (21) to post: 124 (32)). All other positions showed solely a time effect (*p* < 0.001) but no group × time interactions (0.23 < *p* < 0.76).

### 3.5. Perceived Exertion

No significant side × position × group interaction was found for subjective perceived exertion levels after the 12 consecutive trials (F4,23 = 0.40, *p* = 0.53, η_p_^2^ = 0.015). Post exercise, RPE values increased to: left-sided, bottom recreational: 13.8 (SD 1.5); ambitious: 14.6 (2.1), *p* > 0.05; left-sided, top recreational: 14.5 (SD 1.6); ambitious: 15.5 (1.4), *p* = 0.43; right-sided, bottom recreational: 14.9 (SD 2.5); ambitious: 14.7 (1.9), *p* = 0.73; and right-sided, top recreational: 14.2 (SD 2.1); ambitious: 14.8 (1.9), *p* = 0.56.

## 4. Discussion

The hypothesis that handgrip decline varies with climbing level can be refuted. To the best of our knowledge, for the first time the present study investigated time-series data of maximal isometric handgrip strength within a climbing-specific work–relief cycle in recreational and ambitious climbers. Thereby, different arm–hand positions for the left and right side were compared in both groups. Mainly maximal isometric handgrip strength differed between the dominant (right) and non-dominant hand as well as between ambitious and recreational climbers in nearly all testing positions. Percentage decrease in grip strength following repetitive maximal grip contractions did not differentiate between groups, though it did between the left and right side for the upper position. Perceived exertion levels and heartrate response did not relevantly differ between groups, sides, or positions.

Compared to age-related norm values of grip strength in younger [20] and middle-aged adults [21], climbers of both levels in the present study showed notably higher absolute maximal grip strength values (at least >15%). With higher force values on the dominant side, the occurring side difference of maximal handgrip is fairly in line with previous findings [22]. However, it has been previously observed that the amount of difference between the left and right side tend to decrease from recreational to elite climbers [23]. Disappearance of asymmetry level has been attributed to a more symmetric load application induced by the training of elite climbers. A more symmetric load application in better climbers during an axial-symmetric climbing tour was corroborative found previously [7]. Interestingly, the present study also revealed that the better climbers showed less pronounced grip strength asymmetry between the left and right side.

Although ambitious climbers showed consistently higher grip strength values compared to the recreational climbers at each contraction cycle, grip strength-endurance capacity did not reveal notable group differences. It seemed more likely that climbing level is mainly determined by maximal isometric strength capacity. Since a comparatively higher maximal strength led to enhanced neuromuscular function, type 2a fiber recruitment and movement economy, it seems also plausible to assume that higher local maximal strength capacities also trigger higher local endurance performance [24] throughout the training process. This is of more importance, since climbing can be regarded as a clear maximal-strength-endurance sport discipline [1,23,25]. Concerning the relationship between maximal grip strength and grip strength-endurance, concordant observations have been made over the last years in healthy young subjects [13,26,27]. These findings might, thus, be transferable to climbers. From a training-related point of view, it appears to be required to first develop a high amount of local maximal strength in climbing-specific muscles (for grip strength, the brachioradialis muscles and the musculus flexor digitorum superficialis) with a subsequent endurance-specific emphasis. As boulder athletes showed higher maximal voluntary contraction (MVC) forces and rate of force development (RFD) compared to climbers [28], high intensity boulder exercises may serve as an appropriate training routine to enhance maximal strength. Endurance capacity can then be trained by extending the required time frames of climbing.

Regarding heartrate and perceived exertion levels, no relevant differences between groups have been observed. Interestingly, the non-dominant (left) lower position led to a steeper increase in heartrate in recreational climbers than in ambitious climbers from the beginning to the end of the twelve repetitive contractions. This specific finding might be attributed to a less pronounced intermittent recovery of the left-hand side of the recreational group. However, this assumption cannot be addressed with certainty within the present approach. Due to the high relative strength demands of both groups, the perceived exertion level did not differ. It seems of interest whether a certain sub-maximal climbing tour (e.g., two degrees below the maximal climbing level) would lead to differences in perceived exertion level between recreational and ambitious climbers.

### Limitations

Some limitations need to be addressed. The threshold between ambitious and recreational climbers can be regarded as arbitrary. The assignment to the groups was conducted according to previous investigations [7]. Additionally, the sample size of groups differed and was lower for the more trained climbers. No statistical differences of training frequencies were found. Thus, the total training volume did not differ adequately between recreational and ambitious climbers. The applied handgrip dynamometer was not able to record the force–time kinetics for each contraction. A comparison of the force–time integral between both groups was not feasible. It could have been likely that the repetitive peak values occurred at the beginning, the middle, or the end of the 4 s contraction. To better distinguish between different force kinetics, future research should focus on the force–time relationship.

## 5. Conclusions

Maximal grip strength clearly distinguished between recreational and ambitious climbers independent of arm position during repetitive maximal grip strength cycles. The percentage decrease in maximal grip strength is not an adequate measure of climbing specific strength-endurance capacity. Since side differences occurred in all outcome measures, maximal grip strength and grip strength-endurance sufficiently address asymmetry level in climbing. Despite asymmetry level being difficult to measure in climbing, it is unclear whether grip strength asymmetry persists in climbing-related testing tasks. The exercise position of handgrip did not relevantly differentiate between groups. Future research should focus on increasing maximal grip strength as a basic performance requirement after climbing versus boulder exercises. Strength-endurance should be better tested employing climbing specific tests. Grip strength tests should also focus on a comprehensive strength testing approach, considering different hold shapes.

### Practical Applications

Maximal grip strength is feasibly applicable to differentiate between ambitious and recreational climbers. Compared to recreational climbers, disappearing handgrip asymmetry might indicate better climbers. The strength decrement index (SDI) does not appropriately reflect grip strength-endurance in climbers, and repetitive strength decline mainly depends on maximal strength. However, the SDI and repetitive strength testing should be mandatorily employed in a therapeutic and diagnostic setting after hand injuries.

## Figures and Tables

**Table 1:** Anthropometric data of the included subjects.

Variable	Recreational (*n* = 17)	Ambitious (*n* = 11)	Total (*n* = 28)
Sex (f/m)	3/14	0/11	3/25
Age (years)	25.1 ± 7.4	30.6 ± 8.2	27.2 ± 8.0
Body mass (kg)	70.2 ± 9.2	72.2 ± 8.2	71.4 ± 8.8
Height (m)	1.78 ± 0.05	1.81 ± 0.09	1.79 ± 0.07
BMI (kg/m^2^)	21.5 ± 2.0	21.8 ± 1.9	21.6 ± 1.9
Ape index	1.06 ± 0.23	1.01 ± 0.03	1.05 ± 0.18
Climbing level (UIAA metric)	6.8 ± 0.5 ***	8.7 ± 0.6	7.6 ± 1.0
Training frequency (times/week)	2.0 ± 0.9	2.5 ± 1.1	2.2 ± 1.0

BMI, body mass index; data are indicated as mean ± standard deviation (SD). Statistical significance level was, as intended, only present for the climbing level (*p* < 0.001 ***).
